# The Neural Correlates of Crowding-Induced Changes in Appearance

**DOI:** 10.1016/j.cub.2012.04.063

**Published:** 2012-07-10

**Authors:** Elaine J. Anderson, Steven C. Dakin, D. Samuel Schwarzkopf, Geraint Rees, John A. Greenwood

**Affiliations:** 1 UCL Institute of Ophthalmology, 11-43 Bath Street, London EC1V 9EL, UK; 2 UCL Institute of Cognitive Neuroscience, 17 Queen Square, London WC1N 3AR, UK; 3 Wellcome Trust Centre for Neuroimaging, University College London, 12 Queen Square, London WC1N 3BG, UK; 4 Laboratoire Psychologie de la Perception, Université Paris Descartes, Paris 75006, France; 5 CNRS UMR 8158, Paris 75006, France

## Abstract

Object recognition in the peripheral visual field is limited by crowding: the disruptive influence of nearby clutter [1, 2]. Despite its severity, little is known about the cortical locus of crowding. Here, we examined the neural correlates of crowding by combining event-related fMRI adaptation with a change-detection paradigm [3]. Crowding can change the *appearance* of objects, such that items become perceptually matched to surrounding objects; we used this change in appearance as a signature of crowding and measured brain activity that correlated with the crowded percept. Observers adapted to a peripheral patch of noise surrounded by four Gabor flankers. When crowded, the noise appears oriented and perceptually indistinguishable from the flankers. Consequently, substitution of the noise for a Gabor identical to the flankers (“change-same”) is rarely detected, whereas substitution for an orthogonal Gabor (“change-different”) is rarely missed. We predicted that brain areas representing the crowded percept would show repetition suppression in change-same trials but release from adaptation in change-different trials. This predicted pattern was observed throughout cortical visual areas V1–V4, increasing in strength from early to late visual areas. These results depict crowding as a multistage process, involving even the earliest cortical visual areas, with perceptual consequences that are increasingly influenced by later visual areas.

## Highlights

► Crowding influences brain activity throughout the early retinotopic cortex ► Modulation increases from early (V1/V2) to late (V3/V4) visual areas ► The progressive influence of crowding parallels the increase in receptive-field size ► These findings suggest crowding is a multistage process

## Results

**Figure 1:**
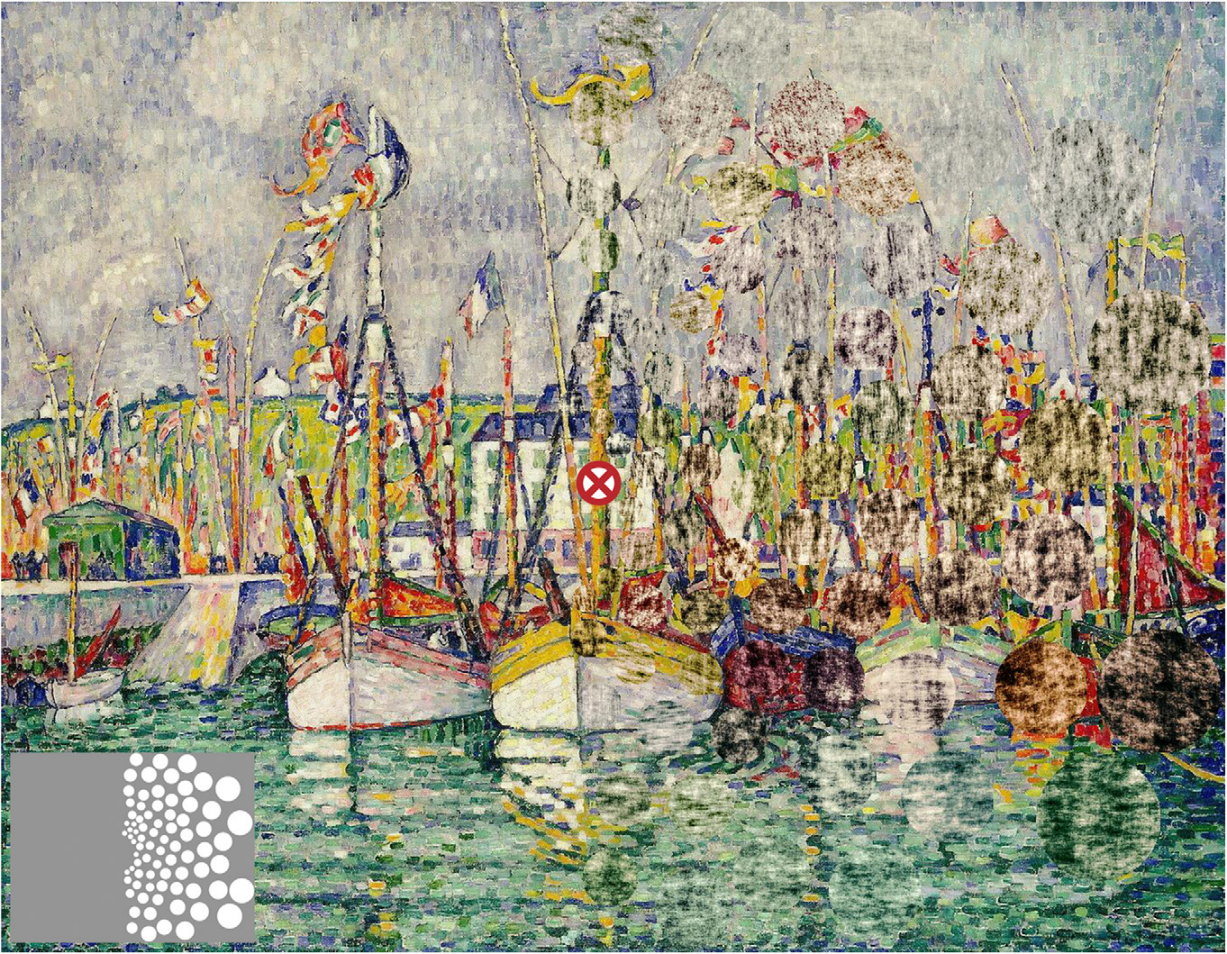
Illustration of Crowding An illustration of visual crowding based on *Blessing of the Tuna Fleet at Groix* by Paul Signac. In the right half of the image, 41% of the pixels have been “phase scrambled” within localized circular patches, preserving only the local contrast or luminance structure at these locations (as indicated in the lower left inset). However, when fixating the central red cross, the scrambled regions become indistinguishable from their surrounding context. For instance, the vertically oriented structure among the boat masts is remarkably similar in the left and right visual fields, despite the right-hand side being considerably disrupted. These changes in the appearance of cluttered visual scenes result from crowding [1, 2], which simplifies the peripheral visual field into texture [3, 14].

Crowding (see Figure 1) is a cortical phenomenon, occurring beyond the level of monocular cells in cortical visual area V1, as dichoptic presentation (target presented to one eye, flankers to the other) does not reduce the effect [4, 5]. Initial evidence placed crowding as a late-stage integration process, occurring after the initial extraction of features [6], as contrast-detection thresholds [7] and orientation-selective adaptation [8] are typically not affected. However, these findings do not hold under all stimulus conditions [9–11], and mounting evidence now suggests that at least some of the neural events associated with crowding must occur at an early stage of processing, such as V1 [12]. Consistent with an early locus, the strength of crowding is critically dependent on the cortical distance between targets and flankers in V1 [13]. However, images containing textured regions (with statistical properties that match the portion of scene they replace) become indistinguishable from one another only when the size of the region approximates the estimated size of V2 receptive fields [14]. In short, psychophysical investigations have done little to constrain the neural basis of crowding.

The few neurophysiological investigations of crowding do little to clarify the psychophysical findings. V4 neurons appear to respond in a manner consistent with the effects of temporal crowding [15] and have receptive-field sizes that fit with the general rule that crowding occurs when the separation between target and distractors is within one half of the target eccentricity [16, 17]. However, V4 lesions appear to have little effect on crowding [18]. Additionally, the neural changes associated with strabismic amblyopia, a condition that induces strong foveal crowding [2, 19], occur primarily within V1 [20].

Similarly few attempts have been made to identify the locus of crowding in humans through the use of neuroimaging [21–24], but findings to date suggest that crowding exerts an influence on blood oxygen level-dependent (BOLD) responses beyond V1, becoming manifest in V2 and remaining evident through V3 and V4. Thus, the balance of evidence suggests that crowding could act at multiple stages of visual processing, a possibility consistent with the effect of crowding on a range of visual modalities [25–28], and on individual features as well as whole objects [29–31].

Previous attempts to identify the cortical locus of crowding have typically involved a physical change to the stimulus in order to induce crowding, such as the introduction of flankers. In such cases, it is difficult to tease apart modulations in brain activity in response to the changed percept (the result of crowding) from modulations in response to the change in stimulus (the addition of flankers). Here, we exploited our earlier finding [3]—that crowding changes the appearance of objects—in order to measure brain activity that correlated with the crowded percept, independent of any change to the surrounding stimulus configuration. Using high spatial resolution, high-field fMRI, and an event-related adaptation paradigm, we showed that crowding exerts an influence on neural responses throughout the early retinotopic visual cortex, with increasing effect from early to late visual areas.

**Figure 2:**
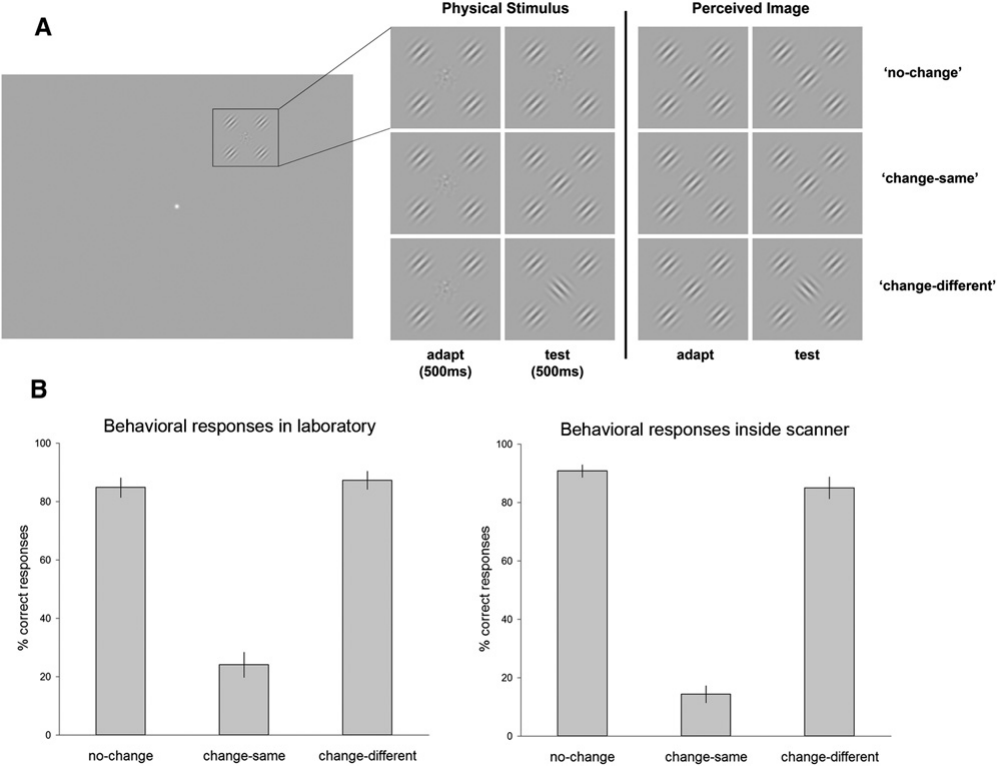
Crowding Stimulus and Behavioral Results (A) Participants maintained central fixation while monocularly viewing a peripheral stimulus, centered at 10° in the upper right quadrant. Each trial began with an adapting stimulus (central noise and four Gabor flankers) for 500 ms, followed by a test stimulus for 500 ms in which the central noise patch may or may not have changed to a Gabor patch. In equal proportions, the noise patch either remained as noise (no-change), changed to a Gabor that matched the orientation of the flankers (change-same), or changed to a Gabor with orthogonal orientation to the flankers (change-different). The change from noise to Gabor always occurred midway through the counterphase time course when all elements reached mean luminance (zero contrast). Participants then had 2,500 ms to indicate (by key press) whether they had detected a change in the central patch or not. An equal proportion of null trials (condition 4), in which no peripheral stimulus appeared, were also included—these were used as a baseline in the event-related fMRI experiment. There were 40 trials of each of the four conditions per experimental run. Flanker orientation remained consistent throughout a trial at either 45° or 135°, presented equally often and in random order. Trial presentation order was generated with an M sequence to ensure that trials of each type were preceded equally often by trials of each of the other types, including itself. In the right half of this figure there is a schematic of how the stimulus appears to those who experience crowding; i.e., what we refer to as the “crowded percept.” Under crowded conditions, the central noise patch appears oriented to match the surrounding flankers. In this situation, switching the target noise patch for a Gabor that matches the flankers goes undetected (change-same), whereas switching the noise patch for a Gabor orthogonal to the flankers is easily detected (change-different). Hence, fMRI responses that follow the crowded percept are expected to show repetition suppression for both the no-change and undetected change-same trials, but release from adaptation in detected change-different trials. (B) All participants performed one run of the behavioral task outside the scanner (40 trials of each condition) to ensure they reached our criterion for inclusion in the fMRI experiment (see Experimental Procedures). The proportion of trials in which the participant correctly detected whether or not a change had occurred was calculated for each condition. The group mean is presented here; error bars indicate SEM. All participants performed three runs of the behavioral task inside the scanner (a total of 120 trials per condition), replicating the pattern of behavior recorded outside the scanner. On average, no-change and the change-different switch were nearly always correctly detected, whereas the change-same switch was rarely detected.

In an initial behavioral experiment, we identified ten observers who experienced robust crowding and satisfied our criterion for inclusion in the main fMRI experiment (see Experimental Procedures). Observers monocularly viewed a peripheral crowded stimulus positioned at 10° eccentricity, which counterphase flickered at 2 Hz (Figure 2A). All trials started with the same adapting stimulus: a target noise patch surrounded by four Gabor flankers, oriented at 45° or 135°. After 500 ms—midway through the counterphase cycle (when all elements reached mean luminance)—the target was switched for a Gabor that either matched the orientation of the flankers (“change-same”) or was orthogonal to the flankers (“change-different”). In an equal proportion of trials, no switch occurred, and the target noise remained unchanged (“no-change”). Under crowded conditions, the change-same switch was rarely detected (group mean 24% ± 14%), whereas the change-different switch was nearly always detected (87% ± 10%) (Figure 2B). This pattern of performance arose because the Gabor introduced in the change-same condition is perceptually matched to the appearance of the adapting stimulus (wherein the target noise is perceived to have an orientation that matches the flankers), whereas the change-different stimulus appeared markedly different from the adapting stimulus (see Figure 2A). The false-alarm rate in no-change trials (i.e., the proportion of trials in which a change was reported when there was none) was ∼15% (±11%). This pattern of behavior was replicated during the fMRI experiment: change-same switch detected, 14% (±9%); change-different switch detected, 84% (±11%); false alarms, 10% (±6%) (Figure 2B). These results are comparable to those obtained previously with these stimuli [3].

**Figure 3:**
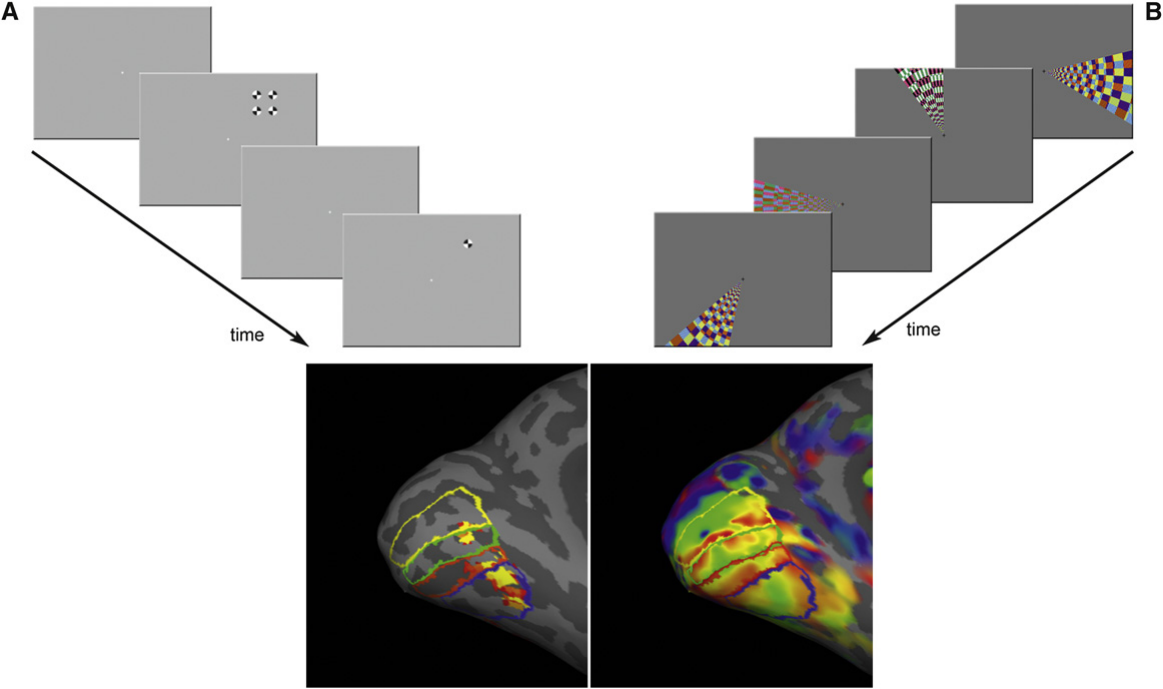
Retinotopic Mapping and Stimulus Localizer All participants underwent two additional scan runs to localize regions of cortex that represent the location of our peripheral stimulus (A), as well as phase-encoded retinotopic mapping to identify the borders between V1, V2, V3, and V4 (B) (see Supplemental Information for full details). (A) Observers maintained central fixation while monocularly viewing blocks of counterphase-reversing black and white checkerboard stimuli positioned to overlap with the location of the stimulus flankers or central target. By contrasting blocks of flanker or target stimulation with blocks of rest, we were able to identify voxels that responded to the location of the four outer flankers or the central noise patch. The data represented here are for the target and flanker locations combined. (B) Observers binocularly viewed a wide-field, flickering, colored wedge stimulus that rotated clockwise or counterclockwise (in separate runs). To ensure that subjects attended to the rotating wedge while maintaining central fixation, a small gray disc appeared at random intervals within the rotating wedge stimulus, and the subject had to report the number of times this occurred in each scan run. The resulting phase map was displayed on a reconstructed inflated surface of the individual's structural scan with FreeSurfer (http://surfer.nmr.mgh.harvard.edu). The boundaries of the visual areas were defined manually by identifying phase reversals in the phase map that corresponded to representation of the vertical and horizontal meridians. Mask volume images were created for ventral V1, V2, V3, and V4 in the left hemisphere for all participants.

Over the course of three functional scanning runs, all participants performed a total of 120 trials of each of the three test conditions—no-change, change-same, and change-different—as well as 120 null trials (see Experimental Procedures). BOLD signal responses to each adapt-test condition were extracted from regions of retinotopic cortex that represented the location of the peripheral visual stimulus (Figure 3) and compared to the fixation baseline (null trials). We predicted that brain areas representing the physical properties of the stimulus would show repetition suppression in no-change trials, but release from adaptation in both change-same and change-different trials (as there was a physical change to the stimulus in both cases). Thus, BOLD signal responses were expected to be comparable for the change-same and change-different conditions, but significantly greater than the response to the no-change condition. In contrast, brain areas reflecting the crowded percept were predicted to show repetition suppression in no-change trials *and* in change-same trials (wherein the target noise was substituted for a perceptually matched Gabor), with release from adaptation occurring only when the target noise was substituted for a perceptually orthogonal Gabor (change-different).

**Figure 4:**
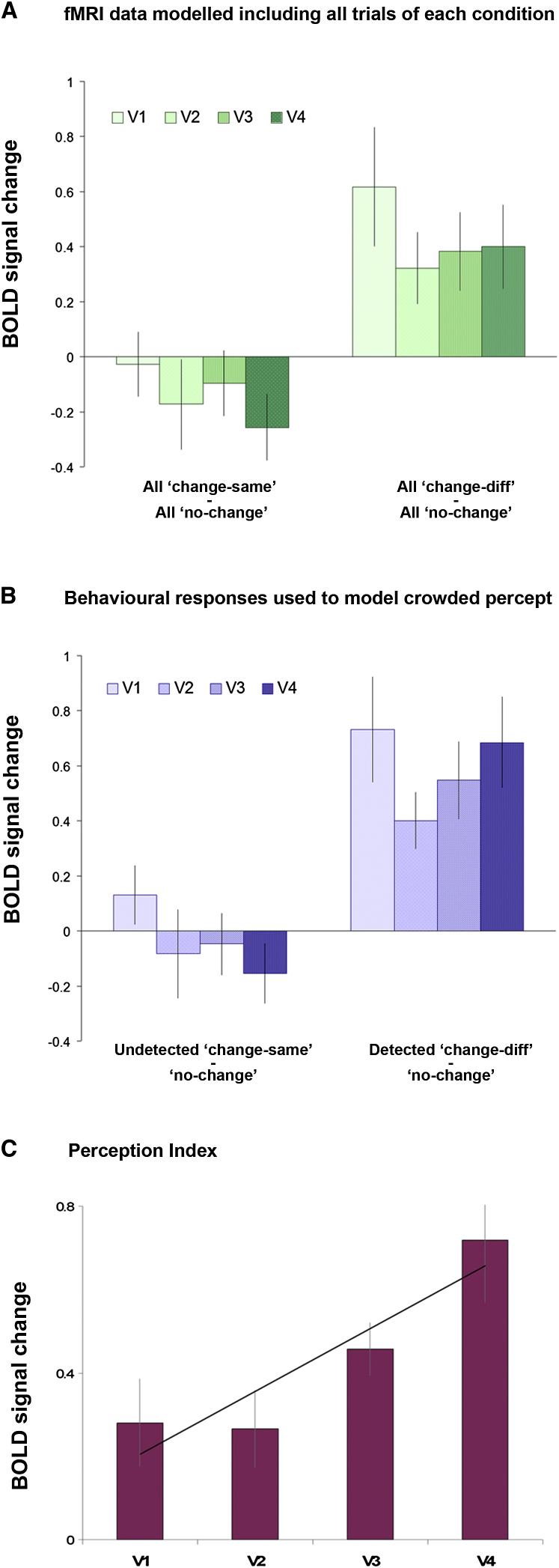
fMRI BOLD Signal Change BOLD signal changes in each adapt-test condition were extracted from voxels of retinotopic cortex that represented the location of the peripheral stimulus. Activity in response to the change-same and change-different conditions has been compared to that of the no-change condition, used as a baseline here for clarity of data presentation. (A) All trials of each condition were modeled, regardless of whether the observer detected a change or not (see Figure S1A). (B) Each individual's behavioral responses were used to model the crowded percept. Only the trials in which the change-same switch was undetected (∼86% of trials) and the change-different switch was detected (∼84% of trials) have been modeled. All false-alarm trials were also rejected (∼10% of trials) (see Figure S1B). (C) We hypothesized that the difference in activity between crowded and uncrowded states would be greatest for brain areas driving the crowded percept. Accordingly, we computed a PI for each visual area by subtracting the activity for crowded trials from the activity for uncrowded trials to yield a single value for each visual area, representing the increase in activity in uncrowded trials compared to crowded trials. Brain areas that preferentially modulate with the crowded percept have a higher (positive) PI, whereas areas more responsive to physical changes in the stimulus have a PI close to zero. There is a clear trend for the crowded percept to be increasingly represented from early to late visual areas. Error bars indicate +/− SEM in all cases.

Initial group analysis found that the pattern of cortical responses in all retinotopically mapped areas, V1 to V4, fit the predictions for a brain area that followed the crowded percept (Figure 4A). That is, repetition suppression occurred in change-same trials at levels comparable to those in no-change trials (V1: t_9_ = 0.155, p = 0.881; V2: t_9_ = 0.788, p = 0.451; V3: t_9_ = 0.535, p = 0.605; V4: t_9_ = 1.439, p = 0.184), and release from adaptation only occurred in change-different trials (V1: t_9_ = −3.022, p = 0.014; V2: t_9_ = −3.556, p = 0.006; V3: t_9_ = −3.524, p = 0.006; V4: t_9_ = −3.173, p = 0.011), and there was also a significant interaction between the two conditions in all areas except V2 (V1: F_2,18_ = 4.786, p = 0.022; V2: F_2,18_ = 2.714, p = 0.093; V3: F_2,18_ = 3.616, p = 0.048; V4: F_2,18_ = 5.829, p = 0.011).

In order to more precisely probe activity that was associated with each individual's percept for each stimulus condition, we used the behavioral responses during the scanning sessions to identify trials in which crowding was present, i.e., when the change-same switch went undetected (on average, 86% of trials), and trials in which crowding was released, i.e., when the change-different switch was correctly detected (84% of trials). We also separated all false-alarm trials (as described above; 10% of no-change trials). This allowed us to model these different responses to trial types independently, providing a closer representation of the crowded percept. Once again, visual areas V1 to V4 all demonstrated the predicted pattern of responses for areas that follow the crowded percept (Figure 4B). That is, repetition suppression occurred in undetected change-same trials, and activity was indistinguishable from that in correctly detected no-change trials (V1: p = 0.444, V2: p = 0.687, V3: p = 0.775, V4: p = 0.358), whereas release from adaptation occurred in correctly detected change-different trials, resulting in significantly greater activity than that for no-change trials (V1: p = 0.004, V2: p = 0.002, V3: p = 0.002, V4: p = 0.001). There was also a significant interaction between conditions in all areas (V1: F_2,18_ = 7.460, p = 0.004; V2: F_2,18_ = 3.664, p = 0.046; V3: F_2,18_ = 7.608, p = 0.004; V4: F_2,18_ = 10.632, p = 0.001). These findings cannot be attributed to a difference in activity elicited by the test stimuli independent of the crowded percept (e.g., if activity were greater for change-different than change-same stimuli), as the results of two control experiments (see Supplemental Information, control experiments A and B, available online) confirm that all three test stimuli (“noise,” “same,” and “different”) evoked comparable activity in the early visual cortex (Figure S2).

Although the pattern of responses illustrated in Figure 4B demonstrates that activity in all early visual areas, V1–V4, followed the crowded percept, there was also an increase in BOLD signal in V3 and V4 when the behavioral responses were used to precisely model each individual's percept. That is, when correctly detected (uncrowded) change-same trials and incorrectly missed (crowded) change-different trials were removed from the analysis (compare Figures 4A and 4B), the difference in V3 and V4 activity in response to change-same and change-different trials increased. This suggests that the activity in these two higher visual areas is more tightly linked to the individual's percept than the activity in V1 and V2.

To demonstrate this more clearly, we calculated a “perception index” (PI) for each visual area, indicating the degree to which the BOLD signal modulated with the crowded percept. We hypothesized that the difference in activity between crowded and uncrowded states would be greatest for those brain areas driving the crowded percept. Therefore, we used the individuals' behavioral responses to model all crowded and all uncrowded trials, regardless of stimulus type. Accordingly, all undetected switch trials, for both change-same and change-different conditions, were considered crowded. Here, crowding was maintained throughout the adapt-test cycle, altering the target appearance such that a consistent orientation was perceived throughout (inducing repetition suppression in brain areas driving the crowded percept). Correspondingly, all correctly detected switch trials, for both change-same and change-different conditions, were considered uncrowded. Here, there was a perceptual change between the adapt and test phases, resulting in release from adaptation. We then subtracted the activity for crowded trials from the activity for uncrowded trials to yield a single value for each visual area, representing the increase in activity in uncrowded trials compared to crowded trials. This gave the following equation, where S represents change-same trials and D represents change-different trials:

$$\text{PI}=\left({\text{S}}_{\text{detected}}+{\text{D}}_{\text{detected}}\right)-\left({\text{S}}_{\text{undetected}}+{\text{D}}_{\text{undetected}}\right).$$

The higher the PI value, the greater the modulation in BOLD response to the crowded percept. In contrast, a brain area representing the physical structure of the stimulus (rather than the percept) should respond equally to crowded and uncrowded trials for either stimulus, and hence produce a PI of zero. Figure 4C clearly shows that the PI is above zero for all visual areas but also increases from V1 to V4. Thus, although all visual areas modulate with the crowded percept, there is also a clear trend for the crowded percept to be increasingly represented from early (V1–V2) to late (V3–V4) visual areas (t_19_ = −2.272, p = 0.035).

## Discussion

Using event-related fMRI adaptation, we measured BOLD signal modulations in early visual cortices that correlated with crowding-induced changes in perception. Our results show that crowding influences neural responses throughout the early visual cortex, starting as early as V1, and increasing in effect from early to late visual areas. These findings do not support the notion that crowding is exclusively a late-stage process, occurring after initial feature detection [6, 8], nor that a single locus is responsible for the perceptual consequences of crowding.

Our results differ from previous fMRI studies on crowding, which did not find an effect on V1 responses [21–24]. We propose a number of contributing factors that might explain this discrepancy. First, we maximized power by using a larger group of observers (n = 10) and ensured that all individuals experienced robust effects of crowding—on average, percent-correct responses reduced by a factor of four under crowded conditions. Previous studies have reported weaker effects (e.g., percent-correct responses reduced from ∼92% to 84%), which, although significant, may have been too weak to evoke a corresponding modulation of V1 [23]. In another study, no modulation of the threshold elevation aftereffect was found under crowded conditions, and accordingly, no modulation of V1 activity occurred [22]. Given that the effects of crowding on orientation-selective adaptation [11] and contrast detection [10] only become apparent when the strength of crowding is maximized, it is likely that modulations in V1 activity are also only apparent when the effects of crowding are maximized. Second, we maintained the focus of attention on the target location throughout the adapt and test phases, as removing attention from the stimulus location decreases BOLD responses to crowded stimuli [22, 23]. Third, we used a task that probed a change in target identity without any change to the surrounding flankers. Previous attempts to identify the cortical locus of crowding have introduced a physical change to the stimulus (the addition of flankers) to induce crowding. In these cases, the experimental design is confounded, and the effects of crowding cannot be distinguished from the effects of introducing the additional flankers.

Our findings suggest that crowding exerts an influence even at the earliest stages of cortical processing (V1), consistent with the fact that crowding-induced changes in appearance are strong enough to evoke the tilt aftereffect [3]. In other words, the orientation perceived in these stimuli engages the same low-level mechanisms that signal physical orientation. That V1 does play some role in crowding is consistent with the numerous neurophysiological [32] and fMRI studies [33–37] that now demonstrate that V1 responses strongly correlate with visual perception.

A number of the above studies also find that perceptual modulations increase with progression through the cortical visual hierarchy, mirroring the bias toward representing the crowding-induced change in appearance observed here. A similar emergence of perceived position is seen throughout the cortex [38], with some effects on the early cortex but the greatest effects occurring beyond V3. Similarly, while V1 responses are predictable from the contrast energy of the stimulus, extrastriate areas are better driven by sparse contour structure [39]. Given that crowding is related to processes of contour integration [40, 41], these results indicate that the effects of crowding may reflect interactions in later visual areas that amplify the contribution of sparse edge structure in natural scenes, effectively simplifying the peripheral field. Furthermore, temporal correlations in activity between early and late visual areas have been shown to decrease under crowded conditions [24].

Though crowding shares many similarities with other contextual modulations, such as masking [6, 7, 10], we know from previous work [3] that the pattern of behavioral responses obtained here could not have arisen due to masking or due to the flankers' inhibiting the introduced Gabor in change-same trials. In particular, when the flankers were rotated to match the introduced Gabor, the same pattern of crowding-induced change in appearance was found. This confirms that observers were comparing the introduced Gabor with their original crowded percept. If suppression or information loss were occurring, as would be predicted by both masking [6, 7] and some theories of crowding [8, 42, 43], performance would have been consistently poor regardless of the orientation of the introduced Gabor. Furthermore, these stimuli have a minimal effect on contrast-detection thresholds, contrary to the predictions of both masking and simple inhibition.

The progressive increase in effect from early to late visual areas observed here parallels the increase in receptive-field size at corresponding eccentricities in V1 to V4. Along these lines, crowding-induced changes in target appearance could arise from the pooling of responses within large receptive fields, resulting in target-flanker averaging to promote perceptual similarity between adjacent regions of the peripheral visual field [3, 44]. Population-based receptive-field size (pRF) mapping in humans has recently corroborated the findings of neurophysiological studies [45] and demonstrated a significant increase in pRF size from V1 to V4, particularly at corresponding eccentricities in the peripheral visual field [46, 47]. Thus, the effects of crowding might gain an accumulating effect from target-flanker pooling across increasingly large receptive fields, though feedback connections are also likely to play an important role [24]. The fact that our crowding effects are maximal in V4 is consistent with the observation that the size of V4 receptive fields [17] matches the general rule that crowding occurs when the separation between target and distractors is within one half of the target eccentricity [16]. V4 may thus be the maximal site of integration, though recurrent connections between V1 and higher visual areas (such as V4) may then act to boost the effect in lower visual areas [48, 49].

## Experimental Procedures

### Participants

Fourteen healthy participants, aged 25 to 42 years, with normal or corrected-to-normal visual acuity, gave written informed consent to take part. Ten participants met the criterion for inclusion in the fMRI study (see next section), and two met the criterion to take part in a subsequent control experiment (see Supplemental Information). All procedures were approved by the local ethics committee.

### Behavioral Experiment

Prior to scanning, all participants performed the behavioral task to ensure they experienced robust crowding and reached our criterion for inclusion in the fMRI study.

#### Stimuli

All stimuli were programmed in Matlab, with Cogent Graphics toolbox used for graphical presentation (http://www.vislab.ucl.ac.uk). A 60 Hz CRT monitor was calibrated and linearized to give a mean and maximum luminance of 50 and 100 cd/m^2^. Stimuli were presented on a uniform gray background, viewed monocularly with the right eye, and centered at 10° eccentricity in the upper right visual field (Figure 2). A central noise patch was constructed from white noise convolved with a log Gaussian filter in the spatial frequency domain. This filtering was isotropic for orientation, with a peak spatial frequency (SF) of 2.5 cycles per degree and a bandwidth of 1 octave. The noise patch was flanked by four Gabor patches, positioned above-left, above-right, below-right, and below-left of the target. Noise and flankers had a center-to-center separation of 2.25°. Gabor stimuli had an SF of 2.5 cycles/deg, a Michelson contrast of 50%, and an orientation at 45° or 135°. The Gaussian windows around both the noise and flankers had an SD of 0.4°. The contrast of all elements counterphase flickered at 2 Hz. Observers maintained fixation on a central white fixation spot throughout. Additional specific task details can be found in the legend to Figure 2A.

Each participant performed one run of the task outside the scanner. The proportion of trials on which the participant correctly responded change/no-change was calculated for each condition. Participants who incorrectly responded “no-change” on at least 60% of change-same trials, correctly responded “change” on at least 60% of change-different trials, and who had no more than 20% false alarms on the no-change condition, were considered to show a reliable effect of crowding and were invited to take part in the fMRI experiment.

### fMRI Experiment

#### Stimuli

Stimuli and trial timing were identical to those used in the behavioral experiment (Figure 2A). Images were projected onto a rear-mounted screen (60 Hz refresh rate), positioned ∼53 cm from the eye, and viewed via a mirror system mounted on the head coil. All images were calibrated and linearized to give a mean and maximum luminance of 50 and 100 cd/m^2^. Participants wore an eye patch over their left eye and responded via a magnetic-resonance-compatible two-alternative button box. All button presses and response times were recorded and saved for offline analysis. Each participant performed three runs of the task inside the scanner. There were 40 trials of each condition per run, hence a total of 120 trials per condition. A new trial presentation order was generated for every scan run, for every participant.

### Scanning Details

#### Main Experiment

All images were acquired with the use of a Siemens 3T Trio MRI scanner with a 32-channel head coil. For the main fMRI experiment, a high-resolution (2.3 × 2.3 × 2.3 mm) echo-planar imaging (EPI) sequence with BOLD contrast (96 × 96 matrix, echo time = 36 ms, acquisition time per slice = 85 ms, repetition time = 2.55, interleaved slice order) was used to acquire 30 near-axial slices, positioned to optimize coverage of the occipital lobe. On every scan run, 228 volumes were acquired, including four dummy volumes at the start of each scan to allow the brain to reach steady state magnetization. A full-brain EPI image (five volumes) was also acquired, with the use of the same acquisition sequence and same slice orientation, for use as an intermediate step in the coregistration process—to improve coregistration of the partial-brain volumes to the full-brain structural image. A high-resolution T1-weighted structural scan was also acquired for every participant.

All participants also underwent a stimulus-localizer scan and phase-encoded retinotopic mapping (see Figure 3 and Supplemental Experimental Procedures for full details).

#### fMRI Analysis

All functional data were preprocessed with SPM8 (http://www.fil.ion.ucl.ac.uk). The dummy volumes from each scan run were discarded, and the remaining images from the main experiment, stimulus-localizer scans and retinotopic mapping scans were realigned and coregistered to the individual's T1 structural image, with the use of the full-brain EPI images and additional structural scan as intermediate steps. All data were then smoothed with a 4 mm isotropic Gaussian kernel.

For the main experiment, in order to identify voxels activated by each of our trial conditions (no-change, change-same, change-different, nulls), a linear combination of regressors was generated, representing the time series for each condition (see Figure S1A). Each regressor was convolved with a synthetic haemodynamic response function. The general linear model, as employed by SPM8, was used to generate parameter estimates of activity for every voxel, for each condition of interest. Data were scaled to the global mean of the time series, corrected for the effects of serial autocorrelations and high-pass filtered to remove low-frequency signal drifts.

In a second model, the behavioral responses of the individuals were taken into account. Seven regressors were generated, representing the time series of correct and incorrect responses for each of the three trial conditions, as well as all null trials (see Figure S1B). With the use of the same methods as above, parameter estimates of activity were calculated for every voxel for each regressor. This model allowed us to extract BOLD signal changes that modulated with the individuals' percept of the stimulus; i.e., to distinguish trials in which a change was detected and those in which a change went undetected due to crowding.

A third model was generated to calculate the PI. Four new regressors were generated: one representing the onsets of correctly detected no-change trials, one representing all trials for which crowding occurred (i.e., when a change was not detected in either change-same or change-different trials), one representing all trials in which crowding was released (i.e., when a change was correctly detected for either change condition), and one representing all null trials. This model was used to compare the BOLD response to all crowded trials and all uncrowded trials, regardless of stimulus type, to give a single value for each visual area—representing the increase in activity in uncrowded trials as compared to crowded trials.

## Supplemental Information

Document S1. Figures S1 and S2 and Supplemental Experimental Procedures, Results, and Discussion
