# Supplementary material for: The Neural Correlates of Crowding-Induced Changes in Appearance
Source: Curr Biol. 2012 Jul 10;22(13):1199–206. doi: 10.1016/j.cub.2012.04.063 (PMC3396841; doi:10.1016/j.cub.2012.04.063)
Supplement: Document S1. Figures S1 and S2 and Supplemental Experimental Procedures, Results, and Discussion [file mmc1.pdf]

Current Biology, Volume 22

Supplemental Information

The Neural Correlates of Crowding-Induced Changes in Appearance

Elaine J. Anderson, Steven C. Dakin, D. Samuel Schwarzkopf, Geraint Rees, and John Greenwood

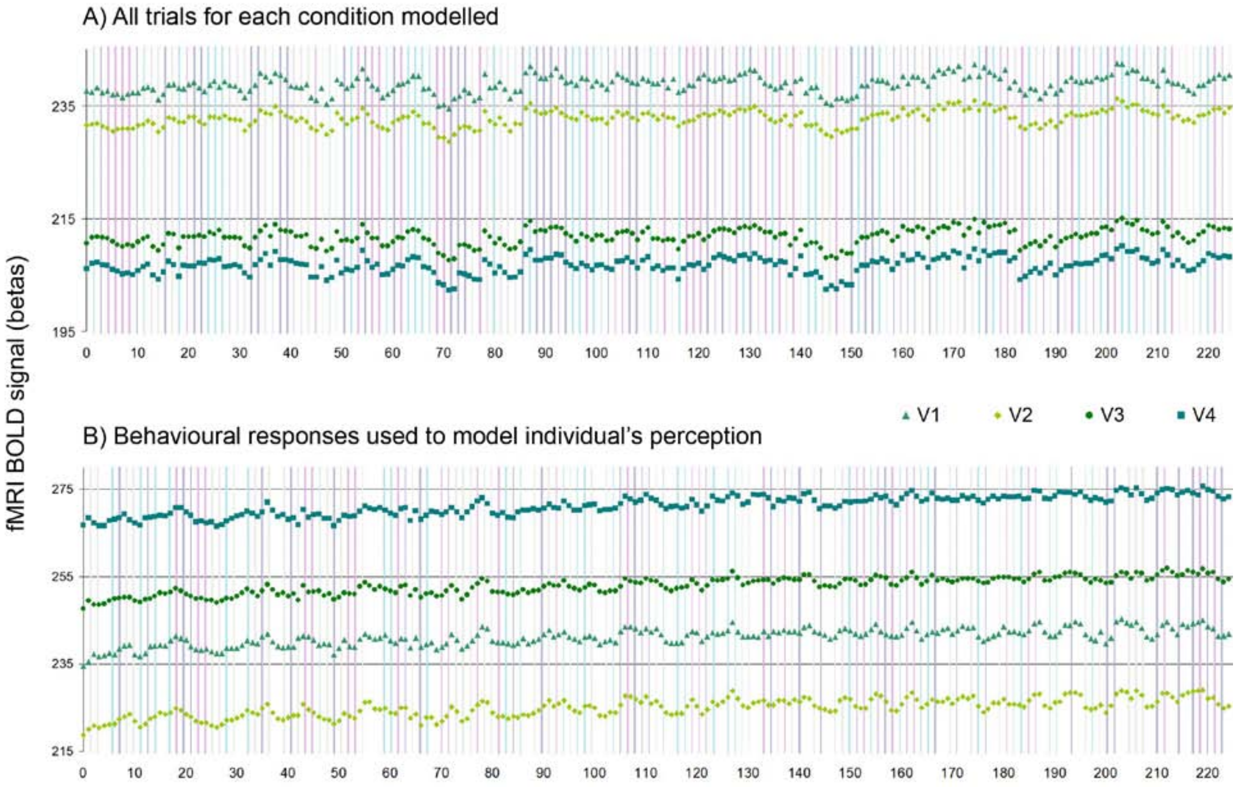

Figure S1. Example fMRI Time Series for Each Region of Interest (ROI), Related to Figure 3

Raw time course data showing changes in BOLD signal for each ROI (voxels representing stimulus location within V1-V4: see Figure 3 and SI methods to follow) for one complete scan run (224 volumes) are shown for two representative individuals from the main fMRI adaptation experiment. In **(A)** the onset of all trials for each condition are indicated by the vertical coloured lines - 'No-change' trials are depicted in turquise, 'change-same' trials in pink, 'change-different' trials in dark blue and 'null' trials in grey. Data from this model type generated the data presented in Figure 4A. In **(B)** only the onset of correct 'no-change' trials (turquoise), incorrect 'change-same' trials (pink) and correct 'change-different' trials (dark blue) are indicated, as well as all null trials (grey), i.e. the individual's behavioural responses have been used to model only those 'change-same' trials on which crowding occurred, and those 'change-different' trials on which crowding was released. Data from this model type generated the data presented in Figure 4B.

Localizing Primary Visual Areas 1–4, Related to Figure 3

In a separate scan session all participants underwent phase-encoded retinotopic mapping to define the cortical visual areas V1 to V4. In order to maximise the visible field of view the front of the head coil was removed. The same scan sequence and slice coverage was used as in the main experiment. Participants binocularly viewed large-field rotating checkerboard wedges (15° width) that fluctuated in both luminance and hue, and extended to the edges of the projector screen (total angular subtense 34°x26°). The wedge either rotated clockwise or anti-clockwise around a small central fixation cross for 10 full cycles (360°). 24 volumes were acquired per cycle (61.2 seconds), giving a total of 240 volumes per polar mapping scan run, plus 5 additional dummy scans. To ensure subjects attended to the rotating wedge, while maintaining central fixation, a small grey disc appeared at random intervals within the rotating wedge stimulus and the subject had to report the number of times this occurred in each scan run. As before, an additional full-brain EPI scan (5 volumes) was acquired in the same session using the same sequence and slice orientation, as well as an additional structural scan (now with the front of the head coil off), to improve co-registration of all functional images to the original structural scan (acquired with the front of the head coil on).

To identify V1, V2, V3 and V4 the time series representing the clockwise and anti-clockwise rotating wedge stimuli were analyzed using a fast Fourier transform to extract the phase and power at the stimulation frequency for every voxel. An F-statistic indicating the significance of the BOLD response was calculated by dividing the power at the fundamental frequency of the stimulus with the average power across all frequencies. The resulting phase map was displayed on a reconstructed inflated surface of the individuals' structural scan using FreeSurfer (http://surfer.nmr.mgh.harvard.edu). The boundaries of the visual areas were defined manually by identifying phase reversals in the phase map that corresponded to representation of the vertical and horizontal meridians (Figure 4). Mask volume images were created for ventral V1, V2, V3 and V4 in the left hemisphere for all participants.

Localizing the Retinotopic Location of the Peripheral Stimulus, Related to Figure 3

An additional functional imaging scan run was used to localise regions of visual cortex that represent the location of our peripheral visual stimulus. The same scan sequence and slice coverage was used as in the main experiment. Using a block design paradigm, observers maintained central fixation whilst passively viewing blocks of counterphase reversing black and white checkerboard stimuli - positioned to overlap the location of the 4 outer flankers or the central noise patch. There were 8 blocks of each stimulus type (central patch or peripheral flankers) interleaved with 16 rest blocks. All blocks lasted 15.3 seconds (6 volumes).

By contrasting blocks of flanker stimulation with rest, or central patch stimulation with rest, we were able to identify voxels that responded to the location of the 4 outer flankers and the central noise patch (Figure 3). For the data presented here, a mask image was created representing activity to both the outer flankers and central patch localiser, thresholded at $p=0.05$ (uncorrected) and combined with each of the V1v, V2v, V3v and V4 mask images.

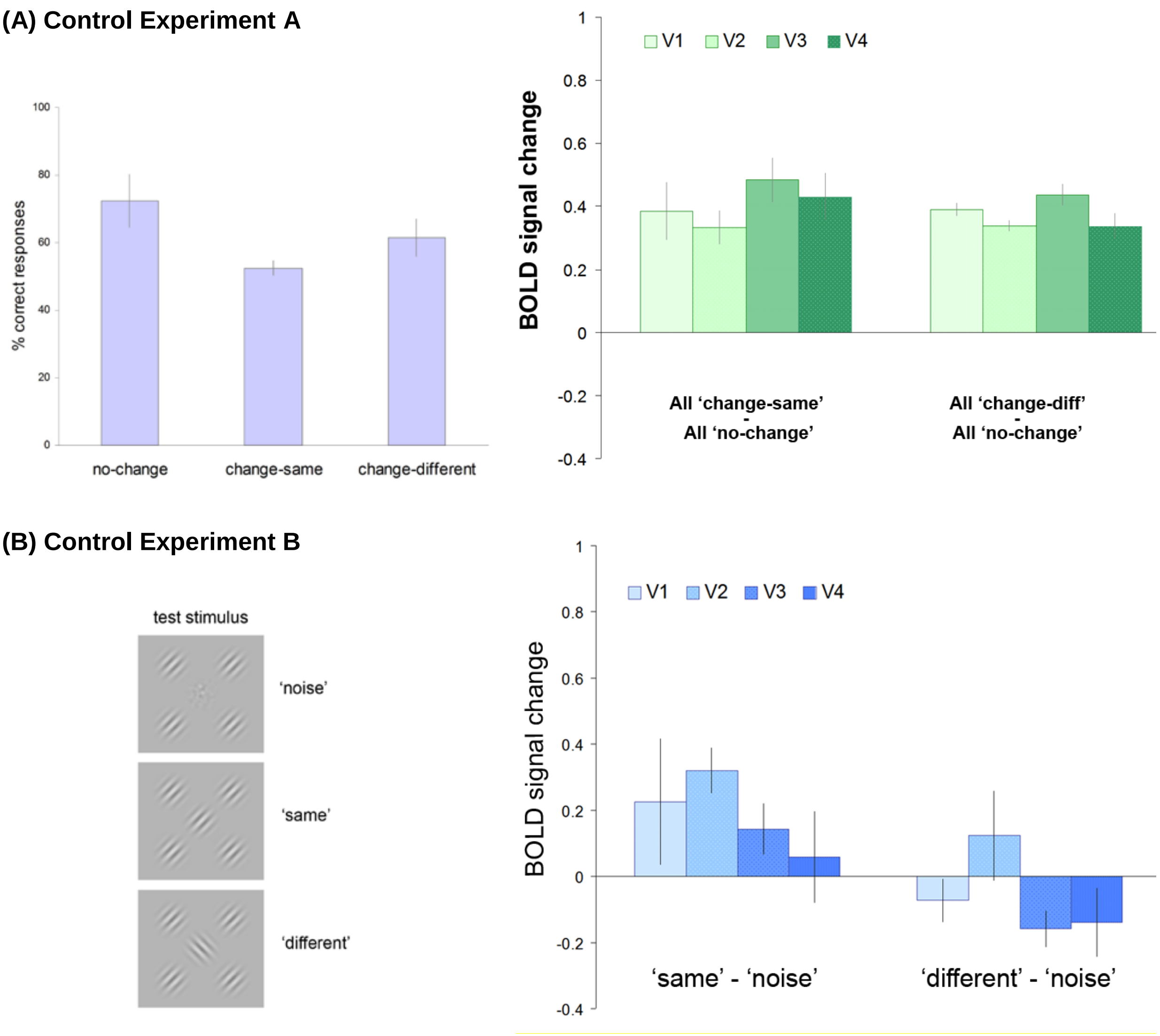

Figure S2. Control Experiments: Assessing Contribution of Stimulus Properties, Related to Figure 4

(A) Two individuals were identified who did not experience robust crowding and had comparable performance on both 'change' conditions. For these two individuals, activity associated with both the 'change-same' and 'change-different' conditions was comparably greater than that for the 'no-change' condition, confirming that for these two individuals both stimulus types evoke comparable activity in all visual areas when crowding is not present.

(B) Six participants from the main experiment took part in an additional control experiment to assess whether the three test stimuli evoked different responses in early visual cortex. For consistency with the main experiment, we used the 'noise' condition as a baseline to compare with the 'same' and 'different' conditions. All three test stimuli evoked comparable activity in all retinotopically mapped areas V1-V4. This confirms that the difference in activity recorded during the main fMRI adaptation experiment was not due to a difference in power between the test stimuli.

Control Experiment A, Related to Figure 4

Participants in the main experiment were selected based on their susceptibility to crowding and our criterion for inclusion in the fMRI experiment (see Experimental Procedures). As a result, for these participants, the 'change-same' trials were rarely detected and the 'change-different' trials were rarely missed (Figure 2B). We chose these criteria in order to maximise power in detecting signal modulations associated with the crowded percept. However, it is possible that the differential response we observe for the two change conditions resulted from the test stimulus itself evoking differential activity in early visual areas, driven by the stimulus properties and not by the crowded percept. The ideal way to test this would be to compare detected (uncrowded) change trials with undetected (crowded) change trials. However, the individual's selected for the main fMRI adaptation experiment experience robust crowding, and hence the 'change-same' switch was rarely detected and the ‘change-different’ switch rarely missed. Therefore, i is not practical to model these few responses and compare the associated BOLD response as there is a gross difference in power between the two conditions (e.g. for the ‘change-different’ condition, 14% of trials were undetected while 84% were detected). Thus, in order to determine whether the two stimuli elicited differential levels of activity in V1-V4, independent of perceptual changes, we identified two further individuals who did not experience robust crowding and thus exhibited less disparity in performance on the two change conditions.

Supplemental Experimental Procedures, Results, and Discussion

These two individuals performed the same fMRI protocol as the main experimental group, as well as retinotopic mapping and stimulus localiser scans. Their data were analysed in the same way as the main experimental group, except that behavioural responses were not included in the fMRI model, as responses were near chance for the two change conditions. The data from these two participants was used as an indicator of whether the stimuli used for the two change conditions elicited different levels of activity in early visual areas, as any difference in activity cannot be attributed to a difference in performance.

The behavioural results are shown in Supplementary Figure 1A, demonstrating that these observers performed around chance on both the 'change-same' and 'change-different' conditions (53% and 62% respectively. This provided us with more comparable numbers of detected and undetected target switches for both 'change' conditions, allowing direct comparison of the two conditions. For these two participants, the pattern of cortical responses differed from that found for the main experimental group. In all retinotopically-mapped areas from V1 to V4, the 'change-same' and 'change-different' conditions evoked comparable activity (Figure S1A). When the 'no-change' condition was used as a baseline for comparison, an analysis of variance, with condition ('change-same - no-change', 'change-different - no-change') and cortical area (V1,V2,V3,V4) as within subject factors confirmed no effect of stimulus type ($F(1,1)=0.349$, $p=0.660$) for any visual area ($F(3,3)=1.378$, $p=0.399$) and no interaction ($F(3,3)=0.235$, $p=0.867$). As the behavioural responses indicated near chance performance, the behavioural responses were not used to re-model the data. However, the contribution from detected and undetected trials was similar for the two conditions and therefore reflects an equal contribution from physical and perceived modulations. These results

confirm that our stimulus did not evoke differential activity in early visual areas due to factors unrelated to perception – in the absence of differential perceptual effects, the physical changes in these stimuli evoked comparable activity throughout V1-V4. Hence, the difference in activity observed for these two conditions in the main experimental group can confidently be attributed to differences in the crowded percept.

Control Experiment B, Related to Figure 4

To further rule out the possibility that the physical properties of our test stimuli evoked differential activity in visual cortex six participants from the main fMRI adaptation experiment also consented to take part in a separate control fMRI experiment. It is conceivable that our test stimuli evoked different responses in early visual cortex based on how much the central target 'popped' out from the surrounding flankers. If so, we would expect greater activity for the 'different' test stimulus when presented in isolation compared with the 'same' stimulus. A second possibility is that the 'change-different' condition may have attracted more attention than the 'change-same' condition due to the salience of the target-flanker difference, as opposed to the matched appearance of the target and flankers in the other two change conditions. Again, were this the case then the 'different' test stimulus should also attract more attention in isolation than the 'same' and 'noise' stimuli and induce greater activity in the same early visual areas. To test these alternative explanations, we measured the BOLD signal response to each of our three test stimuli in isolation. Stimuli were identical to those in the main experiment except that the adaptation phase was removed (replaced by a fixation screen for the same duration) and only the test stimuli presented.

Supplemental Experimental Procedures, Results, and Discussion

The stimuli, timing and fMRI protocols were identical to those in the main experiment except that the adaptation stimulus was replaced with a fixation screen for the same duration (i.e. the timing of each trial remained identical to the original experiment, but only the test stimulus was presented). Instead of detecting changes in the central target between the adapt and test phases, subjects reported if the target patch was the 'same' or 'different' to the flankers. When the central patch was either noise or a gabor oriented orthogonally to the flankers the correct response was 'different', and when the central patch was a gabor oriented to match the flankers the correct response was 'same'. This ensured that attention was directed towards the stimulus throughout the scan run - just as in the main experiment. If the activity recorded in the main experiment was due to a 'pop-out' effect based on the difference between the target and flanker elements in our test stimuli, or to greater attention devoted to the perceived difference in the ‘different’ condition, then we would expect greater activity for the 'different' test stimulus in this experiment than the 'same' stimulus. Data were analysed in the same way as for the main experiment, except that behavioural responses were not included in the fMRI model.

In all retinotopically mapped areas V1-V4 the three test stimuli evoked comparable levels of activity (Figure S1B), confirming that the 'different' stimulus did not evoke greater activity than the 'same' stimulus in visual areas V1-V4. Analysis of variance, with visual area (V1, V2, V3, V4) and test stimulus (noise, same, different), confirmed no main effect of test stimulus ($F=0.821$, $p=0.468$), only

a decreasing trend for a main effect of visual area ($F=3.268$, $p=0.051$), and no significant interaction ($F=0.562$, $p=0.757$). Multiple t-tests to assess differences within each visual area were also performed but no comparisons reached statistical significance.

We can therefore rule out the possibility that the greater activity observed on 'change-different' trials in the main fMRI adaptation experiment was due to the target ‘popping out’ from the flankers in this condition more than in the 'no-change' and 'change-same' trials. We can also exclude the possibility that the difference in activity for these conditions was due to the 'change-different' condition attracting more attention than the 'change-same' condition. Instead, we propose that the difference in BOLD signal reflects different degrees of fMRI adaptation that occurred in response to the perceptual appearance of our crowded stimuli.
